# The utility of adrenal and ovarian venous sampling in a progesterone-producing adrenal tumor and review of the literature

**DOI:** 10.1007/s12020-019-02007-7

**Published:** 2019-08-27

**Authors:** Lian Duan, Yingying Yang, Yu Gu, Xiaobo Zhang, Quanzong Mao, Boju Pan, Chengyan Deng, Hui Pan, Huijuan Zhu

**Affiliations:** 1 Key Laboratory of Endocrinology of National Health Commission, Department of Endocrinology, Peking Union Medical College Hospital, Chinese Academy of Medical Science and Peking Union Medical College, 100730 Beijing, China; 2 Department of Respiratory, Peking Union Medical College Hospital, Chinese Academy of Medical Science and Peking Union Medical College, 100730 Beijing, China; 3 Department of Intervention, Peking Union Medical College Hospital, Chinese Academy of Medical Science and Peking Union Medical College, 100730 Beijing, China; 4 Department of Urological Surgery, Peking Union Medical College Hospital, Chinese Academy of Medical Science and Peking Union Medical College, 100730 Beijing, China; 5 Department of Pathology, Peking Union Medical College Hospital, Chinese Academy of Medical Science and Peking Union Medical College, 100730 Beijing, China; 6 Department of Gynaecology and obstetrics, Peking Union Medical College Hospital, Chinese Academy of Medical Science and Peking Union Medical College, 100730 Beijing, China

**Keywords:** Amenorrhea, Progesterone-producing tumor, Combined adrenal and ovarian venous sampling

## Abstract

**Purpose:**

A clinical case presenting secondary amenorrhea accompanied by an adrenal adenoma and hyperprogesteronemia is described in this study.

**Methods:**

Selective catheterization and sampling of adrenal and ovarian veins were performed.

**Results:**

The source of hyperprogesteronemia was located in the right adrenal gland. A progesterone-producing tumor in the right adrenal gland was diagnosed and removed. Twenty-six days after tumor resection, menstruation occurred.

**Conclusions:**

Progesterone-producing tumors should be considered with the presence of an adrenal mass and hyperprogesteronemia. Combined adrenal and ovarian venous sampling may help to identify the source of progesterone secretion.

## Introduction

Progesterone-producing tumors are extremely rare and easily overlooked in patients with hyperprogesteronemia. To our knowledge, there are only three published reports of adrenal progesterone-producing tumors. We encountered a case that exhibited secondary amenorrhea, elevated serum progesterone levels, and an adrenal tumor suspected to be producing progesterone and related adrenal steroid hormones. We diagnosed the patient’s condition with the use of combined adrenal and ovarian venous sampling. After resection of the adrenal tumor, serum progesterone concentrations soon normalized, and the patient became pregnant. This study is the first to use combined adrenal and ovarian venous sampling to confirm the source of hyperprogesteronemia.

## Methods

### Hormonal measurement

Serum or plasma levels of follicle-stimulating hormone (FSH), luteinizing hormone (LH), progesterone (P), estradiol (E_2_), testosterone (T), β-HCG, prolactin (PRL), dehydroisoandrosterone sulfate (DHEAs), cortisol (F), aderenocorticotropic hormone (ACTH), and urinary free cortisol (UFC) were measured via chemiluminescence immunoassay. Plasma 17α-hydroxyprogesterone (17 α-OHP), aldosterone (ALD), renin activity (RA), and angiotensin (AT) were measured by radioimmunoassay. Plasma and urinary adrenaline, noradrenaline and dopamine were measured using high performance liquid chromatography. Immunohistological analysis of the progesterone was performed using an anti-progesterone antibody (GeneTex, Progesterone antibody, 9B4, 1/50) according to the instructions of the manufacturer. All above procedures were performed at the department of laboratory of Peking Union Medical College Hospital (Beijing, China).

### Combined adrenal and ovarian venous sampling

Combined adrenal and ovarian venous sampling was performed by two experienced interventional radiologists. The patient was placed in the supine position and 5-F vascular catheter was accessed via the right femoral vein. Cobra catheter was then advanced into inferior vena cava and right adrenal vein and Simmons catheter was placed into the left adrenal vein and the right ovarian vein under fluoroscopic guidance, respectively. Cobra catheter was placed into the left ovarian vein afterwards. Five milliliters of venous blood were taken from each site simultaneously for P, 17 α-OHP, E_2_, F, ALD, T, PRA, and a DHEAS assay. The E_2_ ratio between ovarian veins to peripheral vein >2 and the ALD ratio between adrenal veins to peripheral vein >2 was considered as the successful catheterization of the adrenal veins.

### Report of a case

A 33-year-old woman was admitted to Peking Union Medical College Hospital on November 23, 2017 due to a menstrual disorder that had persisted for 15 years. Her first menarche occurred at 12 years old; however, the patient had been suffering from oligomenorrhea since 2002, her last menstruation occurred in October, 2014, and she stated that she had a normal sexual life since get married in 2011 and there was no pregnancy despite no contraception. Her personal and family medical histories were deemed noncontributory to her symptoms. At the start of medical assessment and treatment, she was referred to the Department of Gynecology, and a physical examination indicated she was a young, thin woman (weight: 55 kg, height: 165 cm) with blood pressure varying between 95–120/60–80 mmHg. There was no evidence of Cushing syndrome or virilization.

**Fig. 1:**
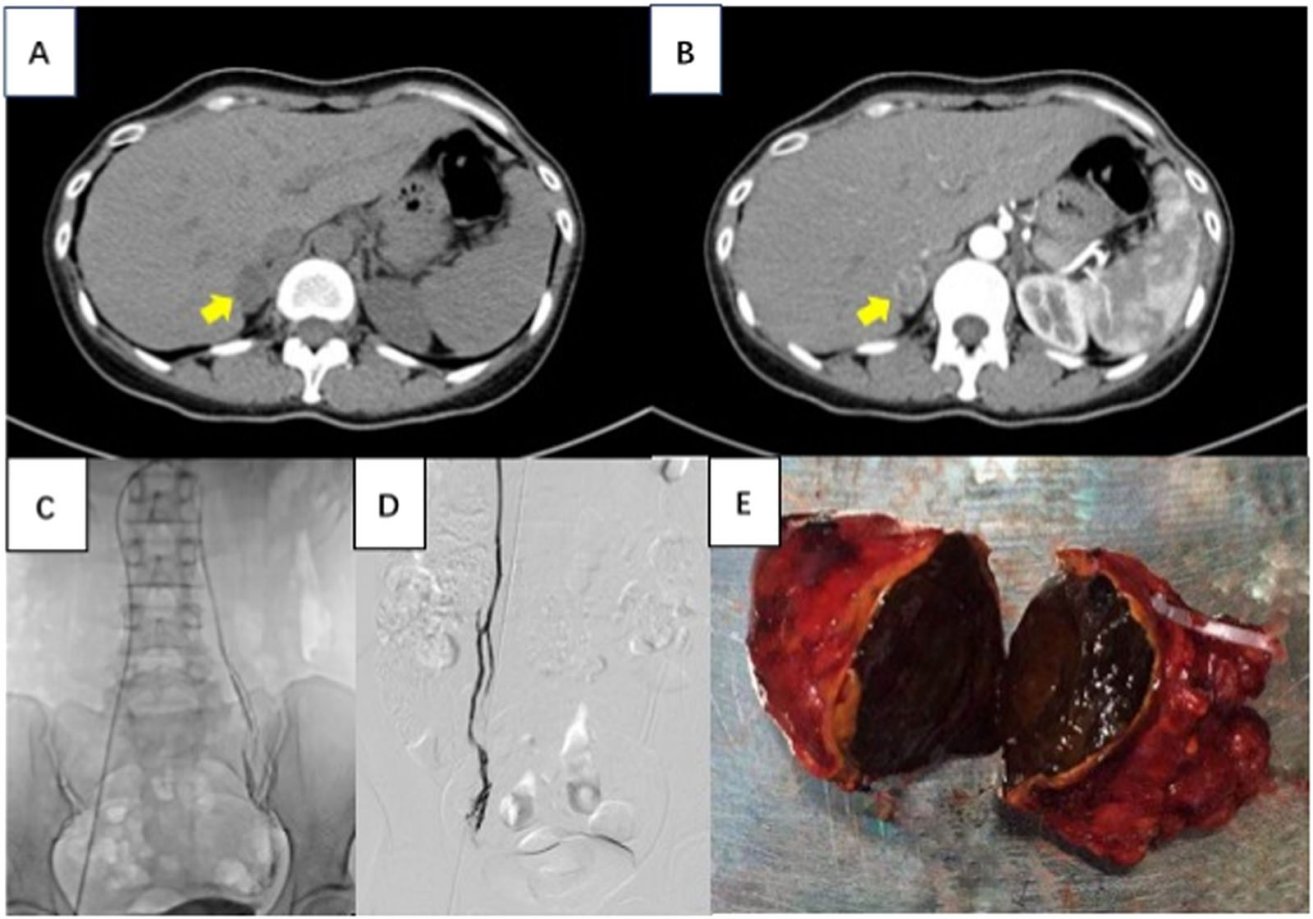
Adrenal CT scan and combined adrenal and ovarian venous sampling. **a**, **b** Adrenal CT scan: the CT shows right adrenal mass, which is heterogeneous, mild enhanced in contrast-enhanced scan (arrows). **c**, **d** Combined adrenal and ovarian venous sampling. **e** Macroscopic appearance of adrenal tumor specimen

A serum steroid test revealed elevated progesterone and 17 α-OHP levels (Table 1). Serum potassium (K) and sodium (Na) levels were normal. This lead to the suspicion of congenital adrenal hyperplasia (CAH) as the cause of the symptoms, and, accordingly, 5 mg of prednisone acetate per day and E_2_ as well as dydrogesterone replacement were prescribed. However, menstruation still did not occur. The patient was then admitted to the Department of Endocrinology, where a medium-dose (3 mg) dexamethasone suppression test, which has been been validated by Zhaolin Lu et al. [1], revealed her plasma progesterone, 17 α-OHP and cortisol were not suppressed whereas her ACTH was suppressed under 5 pg/ml (Table 1). A pelvic ultrasound showed that the patient had a normal uterus and normal ovaries with several follicles. Subsequent computed tomography (CT) of the adrenal glands revealed a 2.8 cm mass with inhomogeneous enhancement in the right adrenal gland. Combined adrenal and ovarian venous sampling was performed. The sampling indicated overproduction of progesterone, 17 α-OHP and cortisol in the right adrenal gland compared to the left gland (Fig. 1 and Table 1). Informed consent was obtained from the patient included in the study.

**Table 1:** Preoperative and postoperative levels of serum and urine steroid hormone concentrations

Plasma hormone	Pre	Post^a^	Plasma hormone	Pre	Post	Urine hormone	Pre	Post
ACTH	23.90	51.20	PRL	17.58	–	24 h UFC (μg/24 h)	63.20	51.60
F (8:00 a.m.)	9.79	5.34	DHEA	59.70	–	24 h UNE	36.49	–
F (0:00 a.m.)	6.14↑	–	PRA	0.83	–	24 h UE	5.45	–
LH	9.29	7.45	AT-II	74.43	–	24 h UDA	275.19	–
FSH	10.41	9.99	ALD	16.20	–			
E_2_	15.00	13.61	TSH	2.48	–			
P	10.75↑	0.25	FT4	1.19	–			
17 α-OHP	19.16↑	0.51	FT3	2.92	–			–
β-HCG	0.40	–						

DST (3 mg)	ACTH (pg/ml)	F (μg/dl)	P (ng/ml)	17 α-OHP (ng/ml)	T (ng/ml)	DHEAS (μg/dl)
Basal values	18.40	8.84	8.92	15.33	0.32	20.1
0.75 mg × 4 Day 2	<5.00	8.24↑	13.02↑	18.67↑	0.22	20.4

AVS + OVS	Peripheral vein	Inferior vena cava	Ovarian vein		Adrenal vein	
			Left	Right	Left	Right
F	6.40	8.18	5.23	6.13	8.87	70.84↑
P	8.60	14.42	9.17	10.07	7.99	641.70↑
17 α-OHP	14.92	29.20	16.26	15.89	17.53	85.65↑
E_2_	62.09	69.34	704.61	1561.68	63.03	48.17
T	<0.10	0.18	0.33	1.48	0.15	1.02
DHEAS	13.50	13.70	13.2	12.8	14.10	16.70
PRA	0.13	0.24	–	–	0.01	0.21
ALD	8.91	9.58	–	–	21.26	32.34

Normal range	Follicular phase	Ovulation	Luteal phase	Menopause
FSH (IU/L)	<10.00	4.54–30.34	1.65–9.66	>40
LH (IU/L)	2.12–10.89	19.18–103.03	1.2–12.86	10.87–58.64
E_2_ (pg/ml)	27–122	95–433	49–291	<40
P (ng/ml)	0.38–2.28	0.93–2.23	5.16–29.26	<0.78
17 α-OHP(ng/ml)	0.1–0.8		0.27–2.90	

Normal range: T 0.1–0.75 ng/ml, DHEAS 23–266 μg/dl, ACTH 0–46 pg/ml, F (8:00 a.m.) 4.0–22.3 μg/dl, F(0:00 a.m.) < 1.8 μg/dl, β-HCG 0–10 IU/l, PRL < 30 ng/ml, DHEA 23–266 μg/dl, PRA 0.93–6.56 ng/ml/h, AT-II 25.3–145.3 pg/ml, ALD 6.5–29.6 ng/dl, TSH 0.38–4.34 μI U/ml, FT4 0.81–1.89 ng/dl, FT3 1.8–4.1 pg/ml, 24 h UFC 12.3–103.5 μg/24 h, 24 h UNE 16.69–40.65 μg/24 h, 24 h UE 1.74–6.42 μg/24 h, 24 h UDA 120.93–330.59 μg/24 h

*DHEAs* dehydroisoandrosterone sulfate, *PRA* plasma renin activity, *ALD* aldosterone, *AT*-II angiotensin, *FSH* follicle-stimulating hormone, *LH* luteinizing hormone, *E*2 estradiol, *P* progesterone, *TSH* thyroid-stimulating hormone, *FT*3 free triiodothyronine, *FT*4 free thyroxine, *UFC* urinary free cortisol, *UNE* urinary norepinephrine, *UE* urinary epinephrine, *UDA* urinary dopamine, *DOC* deoxycorticosterone, *DST* dexamethasone suppression test, *AVS* adrenal venous sampling, *OVS* ovarian venous sampling

^a^Post: 3 days after surgery

## Results

**Fig. 2:**
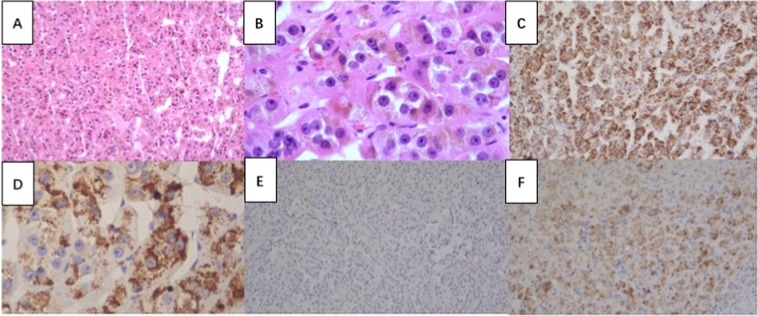
Immunohistochemical-paraffin (IHC-P) findings of the resected adrenal tumor. **a**, **b** Tumor cells were composed of eosinophilic cytoplasm and obvious nucleoli (hematoxylin and eosin, ×100, ×400, respectively) **c**, **d** Progesterone staining using anti-progesterone antibody by IHC-P analysis indicating positive staining of progesterone expression of resected tumor tissue and comparisons with negative control (**e**: adrenocortical adenoma) and positive control (**f**: adrenal cortex-zona reticularis)

The patient underwent laparoscopic resection of the right adrenal tumor, which was suspected of producing the excess progesterone, 17 α-OHP and cortisol (Table 1). A well-encapsulated tumor measuring 2.8 cm × 3.0 cm was removed (Fig. 1E). Unexpectedly, the cut surface of the tumor was an abnormally deep shade of red unique from other kinds of adrenocortical adenoma. The pathology was reviewed by an experienced endocrine pathologist and suggested adrenocortical adenoma. Histologically, the tumor cells were arranged in a nesting and trabecular pattern with eosinophilic cytoplasm and obvious nucleoli. The Weiss score was 2, with a nuclear grade between grade II and III; Only 1% of cells expressed Ki-67. Additionally, <25% of cells were clear, failing to meet the criteria for adrenocortical carcinoma [2]. With adrenocortical adenoma as a negative control, anti-progesterone antibodies detected progesterone immunoreactivity in the tumor cells (Fig. 2). Progesterone and 17 α-OHP levels normalized immediately after resection of the adrenal tumor (Table 1). The patient’s menstruation reoccurred 26 days after resection, and she was pregnant 3 months post surgery.

## Discussion

### Differential diagnosis of amenorrhea

In the presented case, the patient’s menstruation had stopped for more than 6 months despite experiencing prior menstruation. Therefore, secondary amenorrhea, which is often caused by pregnancy, hyperthyroidism, hypothalamic dysfunction, pituitary, or ovarian dysfunction, was considered. The patient’s levels of thyroid hormone, PRL, and β-HCG were within normal ranges, as were her plasma ALD and androgen concentrations. However, elevated plasma progesterone and 17 α-OHP concentrations were found without suppression of LH and FSH. Progesterone is produced by the adrenal glands and by the corpus luteum in the ovaries after ovulation is triggered by LH, but the patient’s serum progesterone levels did not return to normal after the luteal phase. Additionally, ovarian ultrasonography and abdominal CT did not show any abnormal ovarian growths. Thus, elevated progesterone levels of an adrenal origin were suspected. High levels of progesterone and 17 α-OHP are a potential consequence of CAH by 21-hydroxylase or 17-hydroxylase deficiency [3, 4]. CAH was subsequently excluded due to the failure of a medium dose of dexamethasone as well as glucocorticoid administration to normalize hormone levels. With the presence of a mass in the right adrenal gland, the possibility that progesterone, 17 α-OHP and cortisol were autonomously secreted by the adrenal tumor was considered. Since the patient exhibited a favorable clinical outcome to tumor resection, she was diagnosed with a progesterone and related steroid-producing adrenal tumor. It is possible that the patient failed to have artificial menses after she was prescribed estrogen and progesterone due to the endometrium’s poor response to estrogen in the presence of high concentrations of progesterone. However, the mechanism of nonsuppressed plasma LH and FSH at baseline remained unclear. A previous study reported that the increased levels of serum progesterone of adrenal origin may cause an increase in LH concentrations [5]. Though the patient’s cortisol levels were not suppressed by the dexamethasone test and she did not present with any features of hypercortisolemia, suggesting that a diagnosis of subclinical Cushing’s disease cannot be excluded. We observed that ACTH was not suppressed by cortisol excess, and this discrepancy could indicate that the serum cortisol lacked enough biological activity to inhibit that HPA axis.

### Overview of previously reported cases of progesterone-producing adrenal tumors

Progesterone-producing tumors are extremely rare. Previous studies have reported cases with primary amenorrhea revealing an occult progesterone-secreting ovarian Leydig cell tumor and granulosa cell tumors [6–8]. To our knowledge, only three other cases have reported adrenal tumors producing progesterone [9–11] (Table 2). In each previous case, all patients complained of amenorrhea as their first symptom and were found to have elevated progesterone and 17 α -OHP levels. In one patient, serum DOC was significantly increased with coinciding presentation of hypertension and hypokalemia. Giant adrenal incidentalomas over 5 cm in diameter were present in all patients; the large size of the adenomas may be because the presentation of early symptoms in progesterone-producing tumors is not typical. Pathological examination of the tumor tissue after adrenalectomy resulted in the diagnosis of one patient who had an adrenal tumor that measured 12 cm with adrenocortical carcinoma, while the other two cases were diagnosed as benign cortical adenomas. Compared with the previous cases, the maximal diameter of the tumor described in the current study was only 2.8 cm, and no malignant sign was shown by microscopic examination. Elevated adrenal steroid intermediates immediately normalized and menstruation started soon after tumor resection during follow-up.

**Table 2:** Overview of progesterone-producing adrenal tumors

Author	Year	Age	Clinical manifestations	Physical signs	P	17 α-OHP	DST	DOC	T
P. van Zonneveld	1995	18	Amenorrhea	–	18 nmol/l (0.6–2.5)	7.2 nmol/l(<2.2)	Serum cortisol was not suppressed	–	1.8 nmol/l (<2)
Yoshimi Takahashi	2002	21	Amenorrhea	–	8.6 ng/ml (<0.7)	3.5 ng/ml (0.1–3.3)	Cortisol suppressed, but progesterone was not suppressed	0.78 ng/ml (0.03–0.33)	1.0 ng/ml (0.1–0.6)
Masakatsu Sone	2009	27	Hypertension, hypokalemia, and amenorrhea	A palpable abdominal mass	3.53 ng/ml (<0.92)	3.0 ng/ml (0.2–2.8)	DOC and progesterone were not suppressed	8.04 ng/ml (0.03–0.33)	48 ng/dl (9.12–111)
**Author**	**Year**	**Other abnormal steroid concentration**	**Pelvic ultrasound**	**Adrenal tumor size**	**AVS+OVS**	**Surgery**	**Pathology**	**Follow-up**	
P. van Zonneveld	1995	DHEAS 9.1 µmol/L (2–9)	N	6.0–5.5 cm	–	+	keratin (+), Ki-67, P53 (−)	Menstruation occurred 10 days later	
Yoshimi Takahashi	2002	−	N	7*6 cm	The progesterone concentration from the left adrenal vein was 1.45 times higher than right ad-renal vein	+	Various sized cells with eosinophilic granules and polymorphic nuclei	Menstruation occurred 51 days after operation	
Masakatsu Sone	2009	Serum potassium: 2.3 mEq/l; PRA:0.1 ng/ml/h (0.2–2.7); pregnenolone 5.04 ng/ml (0.2–1.5)	N	12 cm	–	+	Nuclear grade was III, mitotic rate was 5 per 50 HPF, necrosis (+), Ki67 > 10%	Blood pressure, serum potassium level normalized, and menstruation occurred 3 months after tumor resection	

### Selective catheterization of adrenal and ovarian veins

Selective catheterization of adrenal and ovarian veins was performed to confirm the source of hyperprogesteronemia and determine whether the right adrenal lesion was functional or nonfunctional. This revealed an over 60-fold increase in progesterone production from the right adrenal gland compared with the left adrenal and bilateral ovarian veins. In addition, 17 α-OHP and cortisol concentrations in the right adrenal gland were 5–7 times higher than those found in the peripheral veins, left adrenal gland, and ovarian veins. Adrenal vein sampling is a highly accurate method used to localize ALD-secreting adenomas. Catheterization of ovarian veins has been used to identify the source of progesterone [6] and selective adrenal venous sampling together with ovarian venous sampling have primarily been used in the localization of occult androgen-secreting ovarian tumors [12–15]. We have also reported the diagnosis of ovarian ACTH-independent ectopic Cushing syndrome with the help of combined ovarian and adrenal venous sampling [16]. The current study, in combination with previous evidence, suggests that selective adrenal and ovarian vein sampling and associated steroid assays may be considered when the source of progesterone and other adrenal intermediate hormones cannot be determined by imaging methods, especially in cases in which the adrenal or ovarian tumor size is not yet large enough to be removed. However, ovarian and adrenal venous catheterization and sampling should only be performed by experienced doctors in order to achieve diagnostic value [12].

## Conclusions

In summary, progesterone-producing tumors are easily overlooked in the differential diagnosis of amenorrhea since they are very rare in routine clinical practice. However, diagnosis of progesterone-producing tumors should be considered with the presence of an adrenal mass; furthermore, ovarian and adrenal venous catheterization and sampling may be highly valuable in the diagnosis of patients presenting small adrenal tumors.

## Supplementary information

Supplementary Information

## References

[1] Tao H, Lu Z, Zhang B, Wang Y, Sun M (2005). Screening nonclassical 21-hydroxylase deficiency in androgen excess women of Chinese Han nationality. Chin. J. Endocrinol. Metab..

[2] Aubert S, Wacrenier A, Leroy X, Devos P, Carnaille B, Proye C, Wemeau JL, Lecomte-Houcke M, Leteurtre E (2002). Weiss system revisited: a clinicopathologic and immunohistochemical study of 49 adrenocortical tumors. Am. J. Surg. Pathol..

[3] Rosenfield RL, Bickel S, Razdan AK (1980). Amenorrhea related to progestin excess in congenital adrenal hyperplasia. Obstet. Gynecol..

[4] Holmes-Walker DJ, Conway GS, Honour JW, Rumsby G, Jacobs HS (1995). Menstrual disturbance and hypersecretion of progesterone in women with congenital adrenal hyperplasia due to 21-hydroxylase deficiency. Clin. Endocrinol..

[5] Eldar-Geva T, Margalioth EJ, Brooks B, Algur N, Zylber-Haran E, Diamant YZ (1998). The origin of serum progesterone during the follicular phase of menotropin-stimulated cycles. Hum. Reprod..

[6] Bry-Gauillard H, Meduri G, Abirached F, Constancis E, Brailly S, Chanson P, Young J (2008). Primary amenorrhea revealing an occult progesterone-secreting ovarian tumor. Fertil. Steril..

[7] Tracy SL, Askin FB, Reddick RL, Jackson B, Kurman RJ (1985). Progesterone secreting sertoli cell tumor of the ovary. Gynecol. Oncol..

[8] Lomax CW, May HV, Panko WB, Thornton WN (1977). Progesterone production by an ovarian granulosa cell carcinoma. Obstet. Gynecol..

[9] van Zonneveld P, Koppeschaar HP, de Bruin TW, Vroom TM, Blankenstein MA, van Vroonhoven TJ (1995). A patient with a progesterone-producing adrenal adenoma who presented with primary amenorrhea. Gynecol. Endocrinol..

[10] Takahashi Y, Ninomiya J, Horiguchi J, Shimizu H, Sato M, Koibuchi Y, Yoshida T, Yoshida M, Takata D, Odawara H, Yokoe T, Iino Y, Morishita Y, Mori M (2002). Primary amenorrhea accompanied by adrenal adenoma: start of menarche soon after tumor resection. Intern. Med..

[11] Sone M, Shibata H, Homma K, Tamura N, Akahira J, Hamada S, Yahata M, Fukui N, Itoh H, Sasano H, Nakao K (2009). Close examination of steroidogenesis disorders in a DOC—and progesterone—producing adrenocortical carcinoma. Endocrine.

[12] Kaltsas GA, Mukherjee JJ, Kola B, Isidori AM, Hanson JA, Dacie JE, Reznek R, Monson JP, Grossman AB (2003). Is ovarian and adrenal venous catheterization and sampling helpful in the investigation of hyperandrogenic women?. Clin. Endocrinol..

[13] Hickman LC, Goodman L, Falcone T (2017). Value of selective venous catheterization in the diagnosis of hyperandrogenism. Fertil. Steril..

[14] Dunne C, Havelock JC (2012). Malignant ovarian Sertoli-Leydig cell tumor localized with selective ovarian vein sampling. J. Minim. Invasive Gynecol..

[15] Petersons CJ, Burt MG (2011). The utility of adrenal and ovarian venous sampling in the investigation of androgen-secreting tumours. Intern. Med. J..

[16] Patel C, Matson M (2011). The role of interventional venous sampling in localising neuroendocrine tumours. Curr. Opin. Endocrinol. Diabetes, Obes..

