# Supplementary material for: The utility of adrenal and ovarian venous sampling in a progesterone-producing adrenal tumor and review of the literature
Source: Endocrine. 2019 Aug 27;66(2):319–25. doi: 10.1007/s12020-019-02007-7 (PMC6838047; doi:10.1007/s12020-019-02007-7)
Supplement: Supplementary file 1 — Supplementary Information [file 12020_2019_2007_MOESM1_ESM.docx]

**Supplementary table 1:** **Sex hormones after surgery**

|  | 5 days after surgery | 11 days after surgery |
| --- | --- | --- |
| FSH (IU/L) | 9.24 | 3.81 |
| LH (IU/L) | 10.79 | 7.81 |
| E2 (pg/ml) | 69.21 | 286.17 |
| P (ng/ml) | 0.43 | 0.32 |
| 17a-OHP(ng/ml) | - | 0.17 |
